# Macrophages at the Crossroads of Chronic Stress and Cancer

**DOI:** 10.3390/ijms26146838

**Published:** 2025-07-16

**Authors:** Sanja Momčilović, Maja Milošević, Dušica M. Kočović, Dragana Marković, Darko Zdravković, Sanja Vignjević Petrinović

**Affiliations:** 1 Group for Neuroendocrinology, Institute for Medical Research, National Institute of Republic of Serbia, University of Belgrade, 11129 Belgrade, Serbia; dusica.kocovic@imi.bg.ac.rs (D.M.K.); dragana.markovic@imi.bg.ac.rs (D.M.); sanja.vignjevic@imi.bg.ac.rs (S.V.P.); 2 Group for Nutritional Biochemistry and Dietology, Center of Excellence for Nutrition and Metabolism, Institute for Medical Research, National Institute of Republic of Serbia, University of Belgrade, 11129 Belgrade, Serbia; mmilosevic@imi.bg.ac.rs; 3 Medical Faculty, University of Belgrade, 11000 Belgrade, Serbia; drdarkozdravkovic@gmail.com; 4 Department of Surgical Oncology, University Hospital Medical Center “Bežanijska Kosa”, 11070 Belgrade, Serbia

**Keywords:** macrophages, stress, tumor, microenvironment, immunomodulation

## Abstract

Macrophages are a heterogenous population of cells that adopt specific phenotypes in response to signals from their dynamic microenvironment. Apart from being key players in innate immunity and in the maintenance of tissue homeostasis, macrophages are also important drivers of low-grade inflammation, which is associated with different chronic conditions including stress and cancer. The activation of macrophages during chronic stress and cancer results in their multifaceted pathogenic roles. Macrophages residing in the tumor microenvironment are commonly known as tumor-associated macrophages and favor or inhibit tumor growth depending on the microenvironmental cues and their activation state. Activated macrophages display a continuum of properties rather than a distinct proinflammatory or anti-inflammatory dichotomy. Emerging evidence suggests that prolonged tissue residency restricts the plasticity of macrophages, while recruited monocytes are more plastic and their differentiation into tumor-associated macrophages during stress can result in a dual imprinting from both the existing stress-induced inflammation and the tumor microenvironment. In addition, the immunomodulation of the tumor microenvironment and reprogramming of tumor-associated macrophages toward the anti-tumor phenotypes have emerged as promising therapeutic approaches. In this review, we will focus on how the persistent inflammatory state underlying chronic stress affects macrophages as well as the macrophages’ contribution to various aspects of tumor growth and progression, highlighting a therapeutic potential of modulation of the macrophage-mediated immunosuppressive tumor microenvironment.

## 1. Introduction

**Figure 1:**
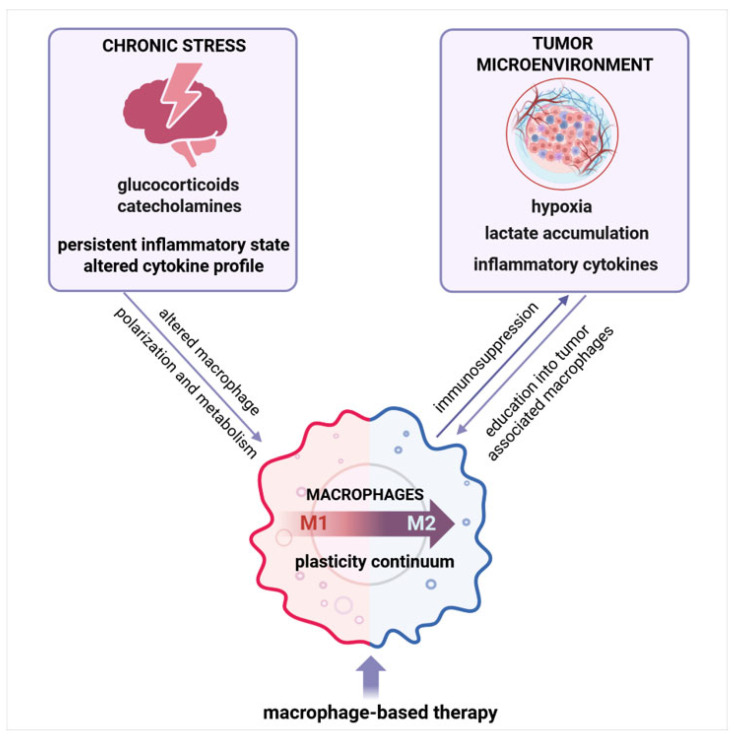
Persistent inflammation underlying chronic stress and cancer shapes macrophages—an integrative overview. Chronic stress and tumor-derived signals shape macrophage polarization and function, driving their differentiation along a plasticity continuum. These factors promote the education of macrophages into tumor-associated macrophages (TAM) with immunosuppressive properties, contributing to tumor progression. Targeting TAM plasticity represents a promising therapeutic approach. Created with BioRender.com, accessed on 10 July 2025.

Macrophages are multifunctional cells that are distributed to virtually all body tissues and contribute to both homeostasis and disease [1]. They are highly plastic cells, capable of adopting distinct phenotypes in response to specific demands of the surrounding microenvironment. The tissue microenvironment is a dynamic milieu composed of both cellular and non-cellular components, which form a regulatory network that helps to sustain the physiological function of an organ [2]. Thus, tissue-specific cells are responsible for the primary function of the tissue, other cells perform supportive functions, while some cells provide information relevant to fate decisions by surrounding cells via directional signaling to these cells. In accordance, macrophages are tissue-resident cells that have the ability to sense the signals released by neighboring cells and transduce these signals into different response outcomes [3]. This ability allows for them to have multifaceted roles, including immune surveillance, phagocytosis, tissue repair, remodeling, and metabolic regulation. Due to these multifaceted roles, macrophages orchestrate various physiological functions that are crucial for homeostatic balance. Understanding how dysregulation of macrophage function contributes to the disruption of homeostasis and a consequent chronic proinflammatory state is essential for the development of strategies to modulate their activity for the therapeutic benefit of different chronic conditions including stress and cancer. Hence, in this review, we discuss how the persistent inflammatory state underlying chronic stress affects macrophages, the role of macrophages in the tumor microenvironment, as well as a therapeutic potential of tumor-associated macrophage (TAM)-centric interventions (Figure 1).

## 2. Macrophages as Orchestrators of Tissue Homeostasis

In their tissue-resident forms, macrophages perform surveillance, constantly monitoring for signs of cellular stress or damage. Once they detect a homeostatic imbalance, macrophages can initiate repair processes by clearing cellular debris and orchestrating the healing response [3]. In order to accomplish the surveillance role, macrophages form intricate networks within tissues that enable them to coordinate their actions and efficiently cover the tissue landscape. Through chemotactic signals, macrophages can direct their movements and actions, creating a highly organized response [4]. In accordance, Taghdiri et al. [5] used a reporter mouse to interpret macrophage cell communication based on correlated cellular calcium dynamics and demonstrated a spontaneous coordination between cells in vivo. The important aspects of macrophage networking are their motility and the physical structure of the tissue. In dense tissues, such as the liver or brain, macrophages, known as Kupffer cells and microglia, are strategically distributed to maximize their surveillance capability. On the other hand, in porous tissues like the lung, macrophages move freely to access a large surface area exposed to the external environment [6]. Beyond interacting amongst themselves, macrophages perform dynamic and adaptable connections with different neighboring cells, like other immune cells, endothelial cells, and fibroblasts. Hence, macrophages cooperate with neutrophils, which exhibit a critical function in tissue repair by promoting the phenotypic conversion of proinflammatory to pro-resolving macrophages [7]. Similarly, the crosstalk between macrophages and fibroblasts is needed for the restoration of normal tissue architecture and effective tissue repair [8]. Moreover, live imaging studies revealed that, after injury, macrophages are attracted to endothelial cells and intimately associate with them during the repair process [9], highlighting the collaborative nature of macrophages in supporting tissue homeostasis.

Beside the networking and collaboration, another crucial aspect underpinning the role of macrophages in homeostasis is their heterogeneity. Tissue-specific macrophages exhibit distinctive transcriptional profiles and functional properties governed by the local microenvironment. This microenvironmental specialization ensures that each tissue type refers to macrophages tailored to its specific needs. Tissue-resident macrophages possess a self-renewal capacity, and the majority of them originate during embryonic development, with minimal contribution from circulating monocytes under steady-state conditions [10,11]. Accordingly, using fate-mapping approaches, Hashimoto et al. [12] demonstrated that lung, splenic red-pulp, peritoneal, or bone marrow tissue macrophages do not arise from monocytes at basal conditions. However, in response to disturbed homeostasis, bone marrow-derived monocytes are recruited to the damaged tissue, where they differentiate into macrophages [11]. More recently, Dick et al. [13] revealed four core markers (CCR2, TIMD4, LYVE1, FOLR2) that can distinguish among macrophages: macrophages with minimal monocyte input, macrophages receiving modest monocyte contribution, and macrophages that are almost entirely replaced by monocytes. Monocyte-derived and tissue-resident macrophages coexist within tissue, and their phenotype over time becomes increasingly similar. In addition, emerging evidence suggests that prolonged tissue residency restricts the plasticity of macrophages, while macrophages derived from newly recruited monocytes retain greater phenotypic flexibility [14]. Moreover, monocyte-derived macrophages during inflammation can be shaped by both the specific traits of the tissue microenvironment as well as the current inflammatory conditions.

## 3. Macrophages in Chronic Inflammation

Due to their ability to polarize into distinct phenotypes with specialized functions, macrophages play a crucial role in the inflammatory response [15]. Historically, macrophages have been classified into two main subsets: M1, or classically activated macrophages, and M2, or alternatively activated macrophages, based on microenvironmental cues and stimuli [16]. Thus, upon exposure to microbial products or proinflammatory cytokines, such as interferon (IFN)-γ and tumor necrosis factor (TNF), macrophages are activated and adopt the M1 phenotype. These cells are potent producers of proinflammatory mediators, including interleukin (IL)-6, IL-1β, Il-12, TNF-α, monocyte chemoattractant protein-1 (MCP-1), and nitric oxide (NO), which enhance pathogen clearance and stimulate adaptive immune responses. By contrast, the M2 phenotype plays a primarily anti-inflammatory and tissue-repairing role. Hence, when an inflammatory response needs to be terminated, niche signals like IL-4 and IL-10 shift macrophages towards the M2 phenotype. M2 macrophages are further subdivided into M2a, M2b, M2c, and M2d depending on the specific cytokines and mediators they produce as well as differentially expressed proteins [16,17]. Although these four subsets differ in phenotype, gene-expression profiles, functions, and cytokine outputs, they share a common hallmark: they produce high levels of IL-10 while generating only low levels of IL-12 [18]. Furthermore, exposure of M1 macrophages to Toll-like receptor ligands (e.g., LPS) and immune complexes suppresses IL-12 and enhances IL-10 production, prompting their shift into the M2b phenotype, designated as regulatory macrophages [19]. M2b macrophages, characterized by Fcγ receptor FcγR crosslinking and high IL-10 and low IL-12 output, secrete IL-4 that skews Th1 toward Th2 cell responses and produce abundant chemokine C-C motif ligand 1 (CCL1), which reinforces their phenotype while recruiting Th2 and Treg cells to establish an immunosuppressive milieu [20].

Nevertheless, recent research has demonstrated that macrophages do not strictly adhere to the M1/M2 classification but rather display a spectrum of activation states [21]. The current concept implies that macrophages can express a variety of intermediate phenotypes depending on the niche signals. In particular, recent advances in single-cell RNA sequencing (scRNA-seq), high-dimensional cytometry, and integrative systems-biology approaches have significantly challenged the traditional view and helped move beyond the M1/M2 paradigm towards a continuum of macrophage activation states influenced by tissue-specific, metabolic, and temporal cues [22,23]. Hence, various niche signals, such as those involving signal transducer and activator of transcription (STAT)1, STAT3/STAT6, and nuclear factor (NF)-κB, guide the macrophage response to specific stimuli, resulting in a broad range of possible activation states [24]. For instance, the activation of STAT1 promotes macrophage polarization toward M1-like phenotypes, presenting with cytotoxic and proinflammatory functions [25]. On the other hand, IL-4- and IL-10-trigered activation of STAT3/STAT6 signaling shifts macrophage polarization to M2-like phenotypes. Similarly, the Toll-like receptor (TLR)/NF-kB (p65/p50) pathway is involved in M1 phenotype programming [26], whereas signaling via phosphatidylinositol 3-kinases (PI3K)/Akt1 and transforming growth factor (TGF)β/SMAD favors the M2 phenotype [27,28]. Furthermore, microRNAs, including miR-155 and miR-223, also have a role in macrophage polarization, with miR-155 being a critical factor in driving macrophages towards the M1 state [29].

The transition between M1 and M2 states is critical in managing inflammation [30]. Disruption in this transition can lead to chronic inflammation, as lingering M1 macrophages may sustain an inflammatory environment. In particular, the balance between STAT1 and STAT3/STAT6 activation within the microenvironment may orchestrate the expression of proinflammatory vs. anti-inflammatory pathways, thus determining macrophage function along the continuum [24]. Dysregulation of cytokine signaling, due to either insufficient levels of anti-inflammatory mediators or impaired receptor function, can inhibit the activation of STAT3 and STAT6 transcription factors, thereby preventing the induction of M2 characteristics. Additionally, persistent activity of NF-κB signaling may dominate the local microenvironment, suppressing M2-associated signaling cascades and thereby maintaining macrophages in a proinflammatory state. Furthermore, chronic overexpression of miRNA-155 has been linked to the persistence of M1 macrophage phenotypes and proinflammatory responses [31]. In contrast, disruption in the expression of miRNA-146a, as a negative feedback regulator that modulates macrophage TLR signaling involved in inflammation, can lead to an imbalance in the negative feedback loop, thereby contributing to excessive inflammation and chronic inflammatory conditions [32].

## 4. Chronic Stress and Macrophages

**Figure 2:**
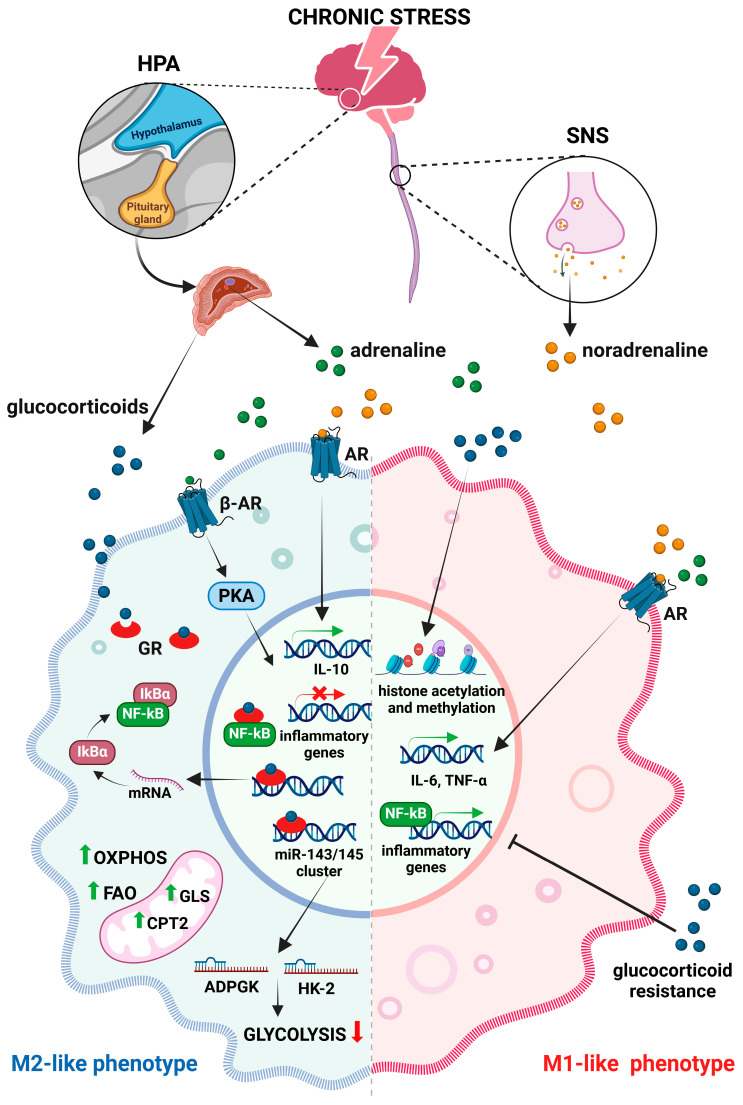
Effects of chronic stress on macrophages. Chronic stress triggers the release of glucocorticoids and catecholamines via activation of the hypothalamic–pituitary–adrenal (HPA) axis and the sympathetic nervous system (SNS), which influence macrophage polarization and the expression of inflammatory genes. Glucocorticoids suppress nuclear factor kappa B (NF-κB) and reduce the expression of glycolytic enzymes (HK2-hexokinase-2 and ADPGK—ADP-dependent glucokinase), leading to a metabolic shift in favor of fatty acid oxidation (FAO) and oxidative phosphorylation (OXPHOS). Catecholamines that bind to adrenergic receptors (AR) promote M1 polarization, leading to sustained production of proinflammatory cytokines and activation of stress-responsive transcription factors. Continuously activated β-adrenergic receptors (β-AR) can also induce anti-inflammatory pathways (like cAMP-phosphatidylinositol kinase A—PKA cascade) and modulate gene expression towards an M2-like phenotype (abbr. CPT2-carnitine palmitoyl transferase 2, GLS-glutaminase, IκB-inhibitors of kappa B). Created with BioRender.com, accessed on 17 December 2024.

Stress has profound impacts on the immune system [33], especially on macrophages, by altering their phenotype and function (Figure 2). In response to stress, the sympathetic nervous system (SNS) and the hypothalamic–pituitary–adrenal (HPA) axis are activated, consequently releasing catecholamines and glucocorticoids that exert both direct and indirect effects on macrophages [34]. While acute stress can have beneficial effects by enhancing the responsiveness of macrophages and boosting their proinflammatory activities [35], chronic stress induces a state of permanent macrophage activation, which leads to a constant production of proinflammatory mediators [36]. This ongoing, long-term inflammation contributes to immune dysregulation and the development of chronic inflammatory diseases including cancer [37]. A stress-induced chronic inflammatory environment stimulates the secretion of glucocorticoids and creates a milieu conducive to alternative macrophage activation, characterized by M2-like phenotypes. Glucocorticoids have been shown to directly affect macrophage polarization by modulating inflammatory gene expression through different mechanisms involving microRNAs and metabolic changes. Thus, cortisol induces the expression of the miR-143/145 cluster that plays an important role in reprogramming macrophage metabolism, favoring the M2-like polarization pathways [38]. In particular, upregulated expression of the miR-143-3p/miR-145 cluster reduces the expression of the glycolytic enzymes hexokinase-2 (HK2) and ADP-dependent glucokinase (ADPGK) while inducing the expression of carnitine palmitoyltransferase 2 (CPT2) and glutaminase (GLS), key enzymes in fatty acid oxidation and glutaminolysis. Hence, under chronic stress conditions, macrophages undergo metabolic shifts that favor oxidative phosphorylation, which is a metabolic trait of M2-like macrophages, instead of glycolysis that is more typical for M1 phenotypes [39]. Furthermore, glucocorticoids repress NF-κB-driven proinflammatory responses [40], thereby promoting the anti-inflammatory functions of M2-like macrophages. However, over time, the responsiveness of macrophages to glucocorticoids diminishes [33] and, subsequently, leads to a stress-induced glucocorticoid resistance in these cells [41]. Due to glucocorticoid resistance, activated macrophages may promote inflammation despite the presence of anti-inflammatory signals, further exacerbating the underlying proinflammatory state and impairing the stress responses. Apart from affecting the production of proinflammatory cytokines, glucocorticoids also change their gene expression. Changes in histone acetylation or methylation patterns may sustain M1-associated gene expression while suppressing genes necessary for M2 polarization, thus reprogramming macrophages toward a hyperinflammatory state [42].

Beside the increased glucocorticoid release, chronic stress activates the SNS and increases the levels of adrenaline and noradrenaline. Binding to adrenergic receptors on macrophages, these neurotransmitters promote M1 polarization. Persistent adrenergic signaling shifts macrophage function, resulting in sustained production of proinflammatory cytokines, including IL-6 and TNF-α [43]. In addition, the activation of stress-responsive transcription factors such as NF-κB is enhanced under chronic stress, further driving M1 polarization. Nevertheless, continuously activated β-adrenergic receptors on macrophages may also result in the induction of multiple anti-inflammatory pathways, like cAMP-phosphatidylinositol kinase A (PKA), which is known to modulate gene expression towards an M2-like phenotype [44]. Furthermore, noradrenaline downregulates proinflammatory cytokine production by rapidly inducing IL-10 secretion [45]. In accordance, Hu et al. [46] demonstrated that the IL-10/STAT3 axis is robustly activated under chronic stress. This stress-induced altered cytokine profile shapes the immune landscape to promote M2 differentiation.

Along with direct effects on macrophages, chronic stress also affects the communication between tissue-resident macrophages and newly recruited monocytes. Thus, chronic stress activates hematopoiesis [47,48,49,50] and mobilizes monocytes from the bone marrow into blood and target tissues, where they differentiate into macrophages [41]. Moreover, monocytes from both stressed mice and humans display a distinct inflammatory transcriptomic pattern because chronic psychological stress triggers the alteration of chromatin structure and reprograms the transcriptional profile of monocytes, predisposing them toward a hyperinflammatory state [51]. Recruited monocytes exhibit greater plasticity, and their transformation into tissue-resident macrophages can be influenced by both the underlying inflammation and the macrophage microenvironment, resulting in the spectrum of macrophage polarization states. Since stress-induced macrophage priming has the potential to be maladaptive and contribute to the development of chronic inflammatory conditions such as cancer [52], identifying the key regulatory pathways and signals that modulate this continuum is a critical area of ongoing research.

## 5. Macrophages Are Critical Players in Tumor Development and Progression

The tumor microenvironment (TME) is complex and dynamic, and tumor surroundings consist of cellular parts and non-cellular components—extracellular matrix (ECM) and blood vessels [53]. The key cells within the TME are adaptive immune cells (T cells, B cells, and NK cells), innate immune cells (macrophages, neutrophils, and dendritic cells), and stromal cells (vascular endothelial cells, fibroblasts, adipocytes, and stellate cells) [53]. The composition of the TME varies between tumor types and evolves from tumor initiation to progression and metastatic dissemination [53,54].

Macrophages play an essential role within the tumor niche, where they can act as both promoters and suppressors of tumor development depending on the specific signals that they receive from the surrounding niche cells [55]. Tumor-associated macrophages are a heterogeneous population of cells within the tumor microenvironment comprising tissue-resident macrophages that originate from prenatal progenitors and monocyte-derived macrophages, which are largely recruited from the circulation [56]. Monocyte-derived macrophages are more plastic, and their polarization within the tumor microenvironment is highly dynamic [14,57]. Hence, current evidence suggests that the development and origin of TAM play a crucial role in determining their susceptibility to manipulation in vivo, which has significant implications for therapy. In the majority of solid tumors, the predominant population of immunosuppressive TAM arises from circulating Ly6C^hi^ (human CD14^++^) monocytes [58]. These monocytes consistently infiltrate the tumor through CCL2–CCR2-directed chemotaxis and undergo local differentiation. These monocyte-derived TAM exhibit significant metabolic and epigenetic adaptability, allowing for them to quickly adjust their transcriptome, chromatin structure, and energy sources in response to stress-induced factors such as hypoxia, lactate accumulation, or cytokine gradients [59,60]. In contrast, tissue-resident macrophages, populations established from the yolk sac or fetal liver that self-renew throughout life, maintain lineage- and tissue-specific profiles. These profiles limit the range of activation states they can achieve without disrupting organ balance. This epigenetic “gate-locking” makes tissue-resident macrophages relatively resistant to pharmacological repolarization, and attempts to do so can lead to unacceptable on-target toxicity in healthy tissue [61].

**Figure 3:**
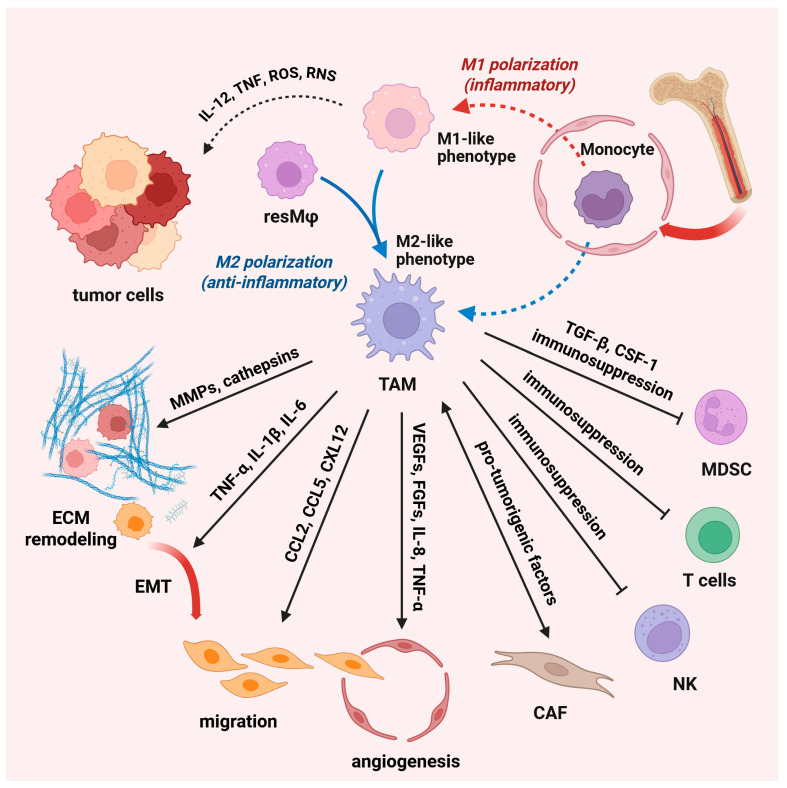
Macrophages in the tumor microenvironment. Macrophages play a crucial role in the tumor microenvironment, as they can both promote and suppress tumor development. Tumor-associated macrophages (TAM) are a diverse cell population that includes tissue-resident macrophages (resMφ) and monocyte-derived macrophages. TAM play a central role in tumor development through immune surveillance, chronic inflammation, and tissue remodeling. In the early stages of tumor development, M1-like macrophages produce proinflammatory cytokines (IL-12, tumor necrosis factor- TNF, and reactive nitrogen—RNS and oxygen species-ROS), while chronic low-grade inflammation leads to tumor development. As the tumor progresses, tumor-derived factors shift macrophage polarization towards an M2-like phenotype and support tissue remodeling, angiogenesis, and immune suppression. TAM secrete matrix metalloproteinases (MMPs) and other proteolytic enzymes (cathepsin) that facilitate tumor invasion and migration. TAM also modulate the behavior of endothelial cells and, thus, promote angiogenic processes. TAM-secreted TGF-β and CSF-1 drive local expansion and immunosuppressive programming of MDSC. TAM can initiate epithelial-to-mesenchymal transition (EMT) through the secretion of pro-tumorigenic factors and upregulate EMT-inducing transcription factors (abbr. IL—interleukin, CCL—chemokines C–C motif chemokine ligand, CXL—stromal cell-derived factor, VEGFs—vascular endothelial growth factors, FGFs—fibroblast growth factors). Created with BioRender.com, accessed on 17 December 2024; modified on 10 July 2025.

Regardless of the origin, TAM play a pivotal role in tumor initiation through their involvement in immune surveillance, chronic inflammation, and tissue remodeling. In the early stages of tumor development, macrophages can exhibit tumoricidal activity. This often corresponds with M1-like phenotypes and the production of proinflammatory cytokines, such as IL-12 and TNF, and reactive nitrogen and oxygen species [56]. On the other hand, chronic low-grade inflammation, mediated by M1-like macrophages, is a hallmark that leads to tumor development [62]. As the tumor progresses, tumor-derived factors shift macrophage polarization towards M2-like phenotypes, which support tissue remodeling, angiogenesis, and immune suppression, creating a microenvironment conducive to tumor growth (Figure 3). Thus, TAM secrete matrix metalloproteinases (MMP), particularly MMP2 and MMP9, which are crucial for degrading the ECM, thereby facilitating the invasion and migration of tumor cells. In addition to MMP, TAM produce other proteolytic enzymes such as cathepsins that further promote tissue remodeling and invasion [63]. Tumor growth is additionally supported by substantial contribution of M2-polarized cells to tumor angiogenesis [64]. Within the hypoxic tumor microenvironment, M2-like cells secrete a variety of proangiogenic factors including vascular endothelial growth factor A (VEGF-A), fibroblast growth factor (FGF2), TNF-α, IL-8, etc. [55]. These factors stimulate endothelial cell proliferation, facilitate the formation of capillary tubes, and stabilize new blood vessels. TAM, especially those known as Tie2-expressing TAM, are closely associated with endothelial cells and modulate endothelial cell behavior, promoting angiogenic processes [65]. In addition, by modulating the ECM and releasing chemotactic factors including CCL2, CCL5, and CXCL12, TAM foster tumor cell migration and direct them towards blood vessels [66,67,68]. This ability of TAM is critical for tumor metastatic progression. TAM, particularly those exhibiting M2-like phenotypes, may also initiate epithelial-to-mesenchymal transition (EMT) through the secretion of TGF-β, IL- 6, and TNF-α, which activate different signaling pathways (e.g., NF-kB, JAK/STAT3, and β-catenin) and upregulate the expression of EMT-inducing transcription factors, such as SNAIL and ZEB1 [57,69]. Furthermore, TAM engage in a dynamic interaction with cancer-associated fibroblasts that results in enhanced secretion of pro-tumorigenic factors as well as amplified premetastatic functions of both cell types. Apart from interaction with cancer-associated fibroblasts, TAM interact with other immune cells, such as regulatory T cells (Tregs), NK cells, and myeloid-derived suppressor cells, to create an immunosuppressive microenvironment [56]. Thus, TAM release TGF-β and CSF-1 [70,71], which promote the expansion and suppressive function of myeloid-derived suppressor cells [72,73]. Modulation of the TAM-mediated immunosuppressive tumor microenvironment has emerged as a promising therapeutic strategy to enhance immunotherapy effectiveness in cancer [74,75].

## 6. The Role of TAM in Cancer Immunotherapy

In addition to conventional tumor therapy, new therapeutic strategies directed towards specific cells within the TME have been introduced into clinical use for the treatment of various malignancies [53,67]. Since the immune system has a critical role in controlling tumor development and progression as well as the response to therapy, immunotherapy has been developed as a promising approach in cancer treatment. Immunotherapeutic strategies are diverse, with the most prominent examples being immune checkpoint blockade (ICB) and cellular immunotherapy with chimeric antigen receptor T cells (CAR-T) and CAR macrophages (CAR-M) [76,77].

Immune checkpoints play a crucial role in regulating immune responses. Based on the ligand–receptor interactions, they involve multiple signaling pathways directed towards the modulation of an immune response to preserve self-tolerance and avoid tissue damage [78,79,80]. Thus, checkpoints, like programmed cell death protein 1 (PD-1) and cytotoxic T lymphocyte associated protein 4 (CTLA-4) expressed on T cells, elicit inhibitory signals upon binding to its ligands PD-L1 and B7-1/B7-2, respectively, that prevent T cell activation and excessive inflammation [79]. In addition to being expressed on activated T cells, PD-1 is also expressed on the surface of other immune cells, including macrophages, whereas the ligands PD-L1 and PD-L2 are expressed on the surface of many cells, including cancer cells and diverse immune cells [78]. However, cancer cells as well as immune cells in the TME, particularly TAM, overexpress some of the inhibitory checkpoints and contribute to immunosuppression. Therefore, immunotherapy with immune checkpoint blockers (ICB) that inhibit such molecules (either a receptor or a ligand) leads to an enhanced immune response against tumor cells and overcomes immunosuppression [79,80].

T cell-directed antibody-based immunotherapy has shown good results in certain tumors, but the expected beneficial response is lacking in many solid tumors [76,81]. Therefore, attention has been focused on TAM, which are the most abundant cells within the TME and express numerous checkpoints that can be targets in tumor therapy [81,82]. There are multiple mechanisms of checkpoint action in TAM as well as different mechanisms that regulate their expression, which depend on the tumor type, tissue type, dynamic interactions within the TME, and the phenotype of the TAM themselves. The abundance of TAM in the tumor microenvironment and the levels of immune checkpoints in these cells have been shown to have a significant impact on ICB response in cancer patients [54,76].

Macrophages express both PD-1 and PD-L1, which are crucial for developing effective cancer immunotherapy strategies—either T cell and ICB dependent or T cell independent, specific for macrophage function [76,83]. In a study by Gordon et al. [82], it was found that both mouse and human TAM express PD-1. Their results revealed that almost all PD-1^+^ TAM display an M2-like phenotype, while PD-1^−^ TAM mainly manifest an M1-like profile. The PD-1 expression increased on TAM over time in mouse models and with increasing disease stage in primary human cancer but only within the M2 subset. TAM PD-1 expression was negatively correlated with their phagocytic activity against tumor cells, while blockade of the PD-1/PD-L1 axis increased macrophage phagocytosis, with the effect of reducing tumor growth. Furthermore, their results indicated that the majority of PD-1^+^ TAM originated from the circulating monocytes rather than from resident immune cells. Overall, their results signify that PD-1/PD-L1 therapies may function through a direct effect on macrophages [82]. The finding that increased PD-1 expression on TAM modulates their polarization and phagocytic activity has been confirmed in many studies [83,84], and it is associated with a poor prognosis, such as in gastric cancer patients [83]. The interplay in the PD-1/PD-L1 axis is complex and context dependent, and Diskin et al. [84] showed that PD-L1^+^ T cells interact with PD-1^+^ macrophages, inducing STAT6-dependent M2-like polarization, which results in impaired adaptive immunity. However, besides cancer cells, TAM are the predominant cells that express PD-L1 within human tumors, and in some cancers, such as hepatocellular carcinoma (HCC), ovarian small cell carcinoma, and breast cancer, the expression level of PD-L1 in TAM is higher than that in tumor cells [85,86,87]. It has been shown that the expression of PD-L1 in macrophages is correlated with better overall survival in patients treated with PD-L1/PD-1 ICB therapy [88]. Using a myeloid-specific *Pdl1*-*knockout* mouse model, Petty et al. [89] showed that PD-L1 expression on TAM is critical for suppression of intra-tumor CD8^+^ T cell function. Single-cell RNA sequencing analysis of human hepatocellular carcinoma revealed that PD-L1 is mainly expressed on M2 TAM [89]. Similarly, Zhu et al. [90] showed that PD-L1-mediated immunosuppression can be induced by infiltration and M2-polarization of TAM in glioblastoma. In contrast, Wang et al. [91] revealed that PD-L1^+^ macrophages are immunostimulatory and associate with a good clinical outcome in patients with breast cancer, while PD-L1^−^ macrophages are immunosuppressive and associate with poor clinical results.

TAM act as effectors in anti-PD-1/PD-L1 therapy, and after treatment with anti-PD-1 or anti-PD-L1 antibodies, they show enhanced phagocytic activity and activation, thereby re-establishing their anti-tumor function [76]. Furthermore, TAM have the potential to shape the response to ICB therapy. The study by Arlauckas et al. [92] revealed that PD-1^−^ TAM capture anti-PD-1 antibodies from the surface of PD-1^+^ CD8^+^ T cells, mediating the resistance to PD-1 blockade. The mechanism of this action depended both on the Fc-glycan of anti-PD-1 antibody and on Fcγ receptors expressed by host myeloid cells. Moreover, inhibition of Fcγ receptors prevented removal of anti–PD-1 mAb and prolonged its effects on CD8^+^ T cells in vivo. TAM could also impact anti-PD-L1 monoclonal antibody immunotherapy, leading to reduced therapeutic efficacy. It was demonstrated that improvement of anti-PD-L1 cancer immunotherapy could be achieved by functional reversion of pro-tumor TAM toward an anti-tumor phenotype via synthetic glycocalyx-mimicking nanoparticles [93].

It is evident that various cancer types employ different mechanisms to regulate the expression and function of PD-1 and PD-L1 in TAM. In addition to tumor-derived factors, such as exosomes and cytokines, macrophages themselves, the hypoxic TME environment, lactic acid accumulation, cytokines originating from various immune cells in the TME, as well as the conditions caused by tumor therapy, such as chemotherapy, radiotherapy, and CAR-T cell immunotherapy, have a great influence on the expression of PD-L1 on TAM [76].

The discovery that TAM respond to anti-PD-1/PD-L1 therapy, which both boosts their phagocytic activity and activation to restore their anti-tumor function and potentially influences T cell responses to immunotherapy, is crucial for enhancing and optimizing cancer immunotherapy [82,83]. In addition, efforts are being made in the implementation of TAM-targeted therapy, such as the elimination of TAM present in the TME; and inhibition of their recruitment and their reprogramming in combination with ICB immunotherapy as a novel therapeutic approach. There are several clinical trials of PD-1/PD-L1 blockade combined with drugs targeting macrophages summarized in Xu et al. [76].

A set of immune-regulatory receptors on macrophages is involved in “don’t eat me” signaling, which plays a crucial role in regulating phagocytosis, including the engulfment of tumor cells. The signal regulatory protein alpha (SIRPα), found on macrophages, and the surface glycoprotein CD47, broadly expressed across different cell types, form a receptor/ligand pair that is essential for regulating macrophage phagocytosis [81]. Often overexpressed on tumor cells, CD47 interacts with SIRPα on macrophages (a trans interaction) to deliver a “don’t eat me” signal, allowing for tumor cells to evade immune detection [94,95]. Macrophages can be induced to phagocytose tumor cells through SIRPα/CD47 blockade [95,96]. Additionally, CD47 and SIRP-α could be expressed on the same cell, and these *cis* interactions modulate *trans* SIRPα/CD47 functions on macrophages. Inhibition of *cis* interactions may cause hyper-phagocytosis, which should be considered in terms of the efficacy of CD47/SIRP blockade, as it may affect the disruption of both *cis* and *trans* interactions [97]. Relevant clinical trials with CD47-targeting antibodies, antibodies targeting SIRPα, and bispecific antibodies targeting CD47 and other molecular targets are summarized in Qu et al. [95]. Regarding the mechanisms by which chronic stress influences tumorigenesis, it has been identified that an imbalance between pro-phagocytic and anti-phagocytic signals due to chronic psychological stress impairs the macrophage’s ability to clear tumor cells. Specifically, glucocorticoids disrupt the balance between the “eat me” signal receptor, low-density lipoprotein receptor-related protein-1 (LRP1), and the “don’t eat me” signal receptor SIRP-α in macrophages, thereby hindering the effective clearance of tumor cells [94]. Also, ectoenzymes CD39 and CD73, both known to be upregulated in response to stress, convert ATP to adenosine, which impairs antibody-dependent cellular phagocytosis of macrophages and functions as a “don’t eat me” signal, thereby disturbing the phagocytic process and leading to immunosuppression [98]. Tissue-resident macrophages express both CD39 and CD73, while M1 macrophages express lower levels of CD39 and CD73 as compared to the M2 subset [99]. Adenosine influences macrophage polarization by promoting a tolerogenic, pro-tumor M2-like phenotype through interaction with A2A and A2B receptors (A2AR and A2BR). Importantly, M2 macrophages exhibit elevated expression of A2AR, which is a primary target of adenosine signaling [98]. Furthermore, siglec-10, present on TAM, interacts with the glycoprotein CD24 expressed on tumor cells, initiating an inhibitory signal that functions as a “don’t eat me” cue to prevent phagocytosis. It has been revealed that CD24 is a highly expressed anti-phagocytic signal in ovarian cancer and breast cancer, demonstrating the therapeutic potential for CD24 blockade in cancer immunotherapy [100]. Future research may uncover additional “don’t eat me” signals that are currently unidentified, which, based on existing data, represent promising immunotherapeutic targets.

Among different immunotherapeutic strategies, cell immunotherapy based on chimeric antigen receptor (CAR) technology represents a cutting-edge and promising approach in treating various cancers. In recent years, the focus was on T cells. CAR-T cell immunotherapy involves genetically engineered T cells, equipped with synthetic CARs, that can bind to tumor-associated antigens and trigger targeted anti-tumor responses via chimeric antigen receptor domains: extracellular single-chain variable fragment (scFv), a hinge domain, a transmembrane domain, and a cytoplasmic signaling domain(s) [101]. CAR-T therapy has shown remarkable success in treating some hematological malignancies, while the effectiveness of this therapy in the treatment of solid tumors is unsatisfactory. In addition, CAR-T therapy has certain drawbacks and is faced with various interfering factors, such as the immunosuppressive TME, impaired infiltration of T cells into tumor sites, dense extracellular matrix within the TME, and the lack of tumor-specific antigens [77,101]. In accordance, CAR-macrophages (CAR-M) that target solid tumors are potential candidates for overcoming some of these barriers due to their antigenic specificity, infiltrating persistence, and other advantages. Although TAM often display immunosuppressive, anti-inflammatory phenotypes, their phenotypic plasticity allows for them to be modified to exhibit anti-tumor effects. CAR-macrophage technology is likely to be the most promising way to achieve this reprogramming. CAR-M cells effectively target and destroy tumor cells, enhance the recruitment and activation of other immune cells, and exhibit potent tumor-suppressive abilities [77,102,103,104]. The primary advantage of CAR-macrophage therapy over CAR-T for the treatment of solid tumors lies in the diverse anti-tumor mechanisms and diverse principles of CAR-M design [67,105]. Expressing CARs in macrophages has shown considerable promise in both preclinical and clinical studies in cancer immunotherapy [103,106].

## 7. Targeting TAM-Associated Modulation of the Tumor Microenvironment: Focus on Their Functional Reprogramming

Within the tumor microenvironment, TAM are educated to adopt phenotypes that support tumor growth and progression, commonly by facilitating immune suppression. In accordance, diverse strategies have been developed to target macrophages within the tumor niche, focusing on their functional reprogramming and improved anti-tumor immune responses [65]. Reprogramming TAM from the immunosuppressive M2-like to the proinflammatory M1-like phenotypes can enhance anti-tumor immunity. This can be achieved through the activation of TLR or the use of CD40 agonists that enhance M1 activation and cytokine production [107,108]. Small molecules, such as inhibitors of PI3Kγ, have also been found effective in reprogramming TAM and sensitizing tumors to immune-mediated destruction. Additionally, use of colony-stimulating factor 1 receptor (CSF-1R) inhibitors has shown potential in reducing TAM numbers [65]. Furthermore, altering metabolic pathways within TAM can modulate their function. Recent studies have illuminated how metabolic shifts define TAM polarization and function. Hence, rather than depending only on surface markers, TAM utilize metabolic pathways to maintain their pro-tumoral characteristics. M2-like TAM primarily utilize fatty acid oxidation (FAO), oxidative phosphorylation (OXPHOS), and glutaminolysis. In contrast, M1-like macrophages rely on glycolysis, an incomplete TCA cycle, and the accumulation of intermediates such as succinate and itaconate [109]. Metabolites produced by tumors, such as lactate, succinate, and specific amino acids, serve as environmental signals that drive TAM toward M2-like phenotypes. For example, lactate accumulation in the TME stabilizes HIF-1α, upregulates PD-L1, and reinforces the immunosuppressive CD206+ phenotype [110]. At the same time, increased activities of arginase-1 and indoleamine 2,3-dioxygenase (IDO) deplete arginine and tryptophan, thereby suppressing T cell effector functions [111].

In order to disrupt the pro-tumor metabolism of TAM, therapies aim to enhance glycolysis and disrupt OXPHOS. M1-polarizing signals, such as TLR agonists and CD40, activate the PI3K-AKT-mTOR-HIF-1α pathways, thereby promoting glycolysis and the pentose phosphate pathway. CD40 agonists not only increase the expression of costimulatory molecules but also alter TAM metabolism. They promote FAO and glutamine-driven TCA cycle activity to produce acetyl-CoA, facilitating histone modifications that trigger the expression of proinflammatory genes [109]. Moreover, preclinical studies using glutamine antagonists (e.g., JHU083) have shown successful reprogramming of TAM in prostate and bladder tumors, leading to improved anti-tumor immunity through increased infiltration of effector T cells [112].

Since M2-like TAM depend significantly on FAO and lipid processing, inhibitors targeting FAO enzymes or drugs that interfere with lipid uptake through CD36 can restrict both the energy supply and polarization of TAM [113]. Notably, in hepatocellular carcinoma, targeting the E2F1–E2F2–CPT2 and LKB1–AMPK–mTORC1–SREBP pathways alters the lipid metabolism of TAM, helping revert their phenotype [114]. Furthermore, the agents that induce lipid peroxidation can either cause ferroptotic death in TAM or redirect them toward an inflammatory state [115].

Hence, disrupting lipid homeostasis in TAM is a potent strategy to enhance anti-tumor response.

Glutaminolysis promotes M2 polarization by generating α-ketoglutarate (α-KG), which facilitates epigenetic and transcriptional modifications. Inhibiting glutaminase or glutamine transporters lowers α-KG levels, directing TAM toward an M1-like phenotype [116]. Similarly, inhibiting arginase-1 or IDO enzymes replenishes arginine and tryptophan levels, thereby restoring NO production and enhancing adaptive immunity [110].

Importantly, these metabolic interventions that promote glycolytic flux or block fatty acid oxidation preferentially redirect monocyte-derived TAM phenotypes toward an M1-like state, demonstrating their adaptable mitochondrial connectivity. In contrast, tissue-resident TAM maintain oxidative phosphorylation, even under extensive metabolic stress, making them resistant to these small-molecule interventions [117]. Combining TAM metabolic reprogramming with immune checkpoint blockade exhibits synergy: metabolic intervention reverses TAM-mediated immunosuppression, facilitating T cell infiltration and increasing ICI response.

Recently, vitamin D has also emerged as a promising agent to modulate TAM-mediated immunosuppression of the tumor microenvironment, given its multifaceted immunomodulatory properties. There is a growing body of evidence that vitamin D plays a role as an immunomodulator, acting as a biological mediator in stress response [118,119]. Calcitriol, the active form of vitamin D, interacts with the vitamin D receptor (VDR) on TAM, influencing their phenotype and function. The activation of VDR by vitamin D can alter the transcriptional programs within macrophages and boost their immune response. In particular, vitamin D-differentiated macrophages show enhanced expression of genes related to inflammation [120]. In accordance, recent evidence suggests that macrophages are polarized towards M1-like phenotypes after treatment with vitamin D and inhibit the malignant phenotypes of ovarian carcinoma cells [121]. In contrast, vitamin D can also induce polarization of the murine macrophage cell line RAW264.7 towards M2-like phenotypes, especially under high glucose conditions [122,123]. Furthermore, Stachowicz-Suhs et al. [124] demonstrated that calcitriol promotes M2 polarization of TAM in 4T1 mouse mammary gland cancer. Nevertheless, the most recent study by these authors shows that calcitriol decreases both M2 and M1 macrophage markers and exhibits anti-tumor effects in breast cancer patients, further supporting both the immunomodulatory and anti-cancer properties of vitamin D [125]. Moreover, vitamin D can inhibit monocyte recruitment [126] and, consequently, reduce the infiltration of TAM within the tumor niche.

Despite significant progress, several challenges in targeting TAM-associated modulation of the tumor microenvironment persists, including TAM heterogeneity, the activation of compensatory mechanisms, and issues with delivery selectivity.

## 8. Conclusions

Macrophages are essential regulators of tissue homeostasis and immune responses but also significantly contribute to a persistent inflammatory state, which underly multiple chronic conditions including stress and cancer. Despite advancements in understanding macrophage biology, certain unresolved issues remain regarding their roles and regulation under chronic conditions. The classification into M1 and M2 phenotypes does not encompass the full spectrum of macrophage diversity observed in vivo, indicating a need for more refined classification systems based on specific functional markers and responses. Monocyte-derived macrophages exhibit high plasticity, and their differentiation into TAM during stress can result in a dual imprinting from both the ongoing stress-induced inflammation and the tumor niche. This ability makes them a promising target to improve cancer treatment outcomes. Stress-induced adrenergic and glucocorticoid cues are unlikely to simply ‘switch’ macrophages from an M1 to an M2 state. Instead, they bias cells toward discrete programs, such as an IL-10^high^ regulatory state or a lipid metabolic signature, that differentially modulate tumor immunity. Likewise, emerging therapeutic interventions should be evaluated for their capacity to reconfigure macrophage circuits rather than broadly inducing a transition from M1 to M2 polarization states. TAM reprogramming offers a nuanced approach to targeting TAM-associated modulation of the tumor microenvironment. By disrupting the metabolic pathways that sustain immunosuppressive functions, TAM can be redirected from tumor supporters to active participants in immune defense. These strategies, especially when integrated with immune agonists and checkpoint therapies, provide a robust framework for next-generation TAM-centric interventions.

## Data Availability

Not applicable.
